# Host Targeted Activity of Pyrazinamide in *Mycobacterium tuberculosis* Infection

**DOI:** 10.1371/journal.pone.0074082

**Published:** 2013-08-28

**Authors:** Claudia Manca, Mi-Sun Koo, Blas Peixoto, Dorothy Fallows, Gilla Kaplan, Selvakumar Subbian

**Affiliations:** Laboratory of Mycobacterial Immunity and Pathogenesis, Public Health Research Institute (PHRI), New Jersey Medical School, Rutgers Biomedical and Health Sciences, Rutgers The State University of New Jersey, Newark, New Jersey, United States of America; Institut de Pharmacologie et de Biologie Structurale, France

## Abstract

Pyrazinamide (PZA) is one of the first line antibiotics used for the treatment of tuberculosis (TB). In the present study, we have used in vitro and in vivo systems to investigate whether PZA, in addition to its known anti-mycobacterial properties, modulate the host immune response during *Mycobacterium tuberculosis* (Mtb) infection. In vitro we have examined the effect of PZA on cytokine and chemokine release by Mtb-infected or Toll-like receptor (TLR) -stimulated primary human monocytes. In vivo, we have investigated at the transcriptional levels using genome-wide microarray gene expression analysis, whether PZA treatment of Mtb-infected mice alters the host immune response to Mtb infection in the lungs. Here, we report that PZA treatment of Mtb-infected human monocytes and mice significantly reduces the release of pro-inflammatory cytokines and chemokines, including IL-1β, IL-6, TNF-α and MCP-1 at the protein and at the gene transcription levels, respectively. Data from microarray analysis also reveal that PZA treatment of Mtb-infected mice significantly alters the expression level of genes involved in the regulation of the pro-inflammatory mediators, lung inflammatory response and TLR signaling networks. Specifically, genes coding for adenylate cyclase and Peroxisome-Proliferator Activated Receptor (PPAR), molecules known for their anti-inflammatory effect, were found to be up-regulated in the lungs of PZA-treated Mtb-infected mice. Based on the microarray findings, we propose that PZA treatment modulates the host immune response to Mtb infection by reducing pro-inflammatory cytokine production, probably through PPAR- and NF-kB- dependent pathways. In addition, our results suggest that inclusion or exclusion of PZA in the TB treatment regimen could potentially affect the biomarker signature detected in the circulation of TB patients.

## Introduction

Tuberculosis (TB) is one of the leading causes of adult deaths attributable to a single infectious agent in humans. The World Health Organization (WHO) has estimated that 8.7 million new TB cases and 1.4 million deaths occurred in 2011 [1]. Standard treatment of pulmonary TB through Directly Observed Therapy, Short-Course (DOTS) consists of a 2-month, intensive phase with 4 antibiotics: isoniazid (INH), rifampicin (RIF), ethambutol (EMB) and pyrazinamide (PZA) followed by a 4-month continuation phase with INH plus RIF [2]. Sputum culture conversion to negative culture after 2 months of standard DOTS therapy has been shown to correlate with non-relapsing cure and is commonly used as a predictor of response to therapy [3]–[6]. However, sputum culture conversion at 2 months is not always predictive of cure in individual patients [7], [8]. Moreover, sputum culture status is not applicable in the context of extra-pulmonary disease [9]. These limitations emphasize the need for additional surrogate biomarkers of response to TB treatment. Consequently, current clinical research efforts are underway to define host biomarkers, including a number of serum markers that are predictive of response to treatment and/or clinical outcome [10]–[15]. In a recent study on the impact of DOTS on plasma markers in HIV infected and uninfected TB patients, we observed that among 24 molecules evaluated, a significant reduction in the concentrations of IP-10 and VEGF was noted in both patient groups. In addition, striking fluctuations in the concentration of IL-1β, IL-6, IL-1RA, IL-15, IL-17, TNF-α, Eotaxin, FGF-basic and GM-CSF were observed after 2 months of therapy, the point at which treatment shifted from the intensive four-drug phase to the continuation phase with RIF and INH only [16]. As an explanation of these findings, we hypothesized that the removal of PZA and/or EMB from therapy at this time may have contributed to the observed changes in cytokine/chemokine levels in TB patient plasma. Interestingly, a recent in vitro study found that PZA treatment of *Leishmania major*-infected murine cells, including J774 cell line, primary mouse macrophages or bone marrow dendritic cells, induced the up-regulation of cytokines [17]. Thus, in the present study, we tested our hypothesis by exploring the ability of PZA to modulate the host immune response during Mtb infection. Using both in vitro (human monocytes) and in vivo (mouse) Mtb-infection systems, we investigated whether PZA treatment can influence the host immune response, thus potentially contributing to the observed patterns of cytokine/chemokine in the serum of TB patients in response to treatment. Results from our in vitro cytokine measurements and genome-wide transcription analysis of Mtb-infected mouse lungs suggest that PZA treatment modulates the host immune response to Mtb infection by reducing pro-inflammatory cytokine production, probably through an NF-kB-dependent pathway.

## Materials and Methods

### Ethics statement

The UMDNJ Institutional Review Board (IRB) determined that the use of blood (buffy coat) for the in vitro study was exempt (IRB # 0120110022). The animal experiments were conducted as per the Animal Welfare Act guidelines for housing and care of laboratory animals and performed in accordance with the Public Health Service Policy Institutional regulations. Approval for mouse studies was obtained from the Institutional Animal Care and Use Committee (IACUC) at UMDNJ (Approval ID; 071D0810).

### Reagents

All chemicals were purchased from Sigma-Aldrich (St. Louis, MO), unless otherwise stated. Stock solutions of PZA were freshly prepared in distilled water, filtered (0.22 µm) and diluted in cell culture media. PZA was used at final concentrations of 10, 50, 100, 200 and 400 µg/ml for in vitro experiments. TLR agonists, Pam2CSK4 (TLR2/6) and Pam3CSK4 (TLR2/1) (InvivoGen, San Diego, CA) were used at a final concentration of 250 ng/ml and LPS (TLR4) was used at 100 ng/ml, as described earlier [18].

### Bacterial culture

Mtb clinical strains (CDC1551 and HN878) were grown in Middlebrook 7H9 medium supplemented with oleic acid albumin dextrose catalase (OADC) enrichment (Difco BD, Franklin Lakes, NJ), 0.5% glycerol and 0.05% Tween 80 (Sigma-Aldrich, St. Louis, MO) at 37°C to logarithmic phase (OD_540_ =  0.6–0.7) and stocks were prepared as previously described [19]. The numbers of viable mycobacteria were evaluated using the colony forming units (CFU) assay [19].

### Human monocyte infections

Buffy coats from anonymous healthy donors were obtained from the New Jersey Blood Center (East Orange, NJ). Peripheral blood mononuclear cells were harvested by Ficoll-Paque separation; monocytes were isolated by adherence, as previously described [20]. Cells were cultured at 3×10^5^ per well in 24-well tissue culture plates (Corning Inc., Corning, NY) for 24 hours in RPMI 1640 medium (Invitrogen, Carlsbad, CA) supplemented with 10% human AB serum (Gemini Bioproducts, Calabasas, CA). Adherent monocytes isolated from independent donors were treated with PZA at selected concentrations, and simultaneously infected with Mtb at a multiplicity of infection (MOI) of 1∶1 (bacteria: monocytes). Control groups included the following conditions: monocytes cultured in media alone, PZA-treated, Mtb-infected, or TLR-stimulated cells. Determination of the bacillary growth by CFU assay was performed by disrupting the cells by probe sonication and plating serial dilutions in Middlebrook 7H10 agar plates (Becton Dickinson, Sparks, MD) [21]. The effect of PZA on cell viability was evaluated by trypan blue exclusion assay (Life technologies).

### Detection of secreted cytokines by Luminex assay

Supernatants from cultured adherent monocytes were harvested at 24 hours post-treatment, filtered (0.22 µm) and frozen at –80°C for batch analysis. Levels of tumor necrosis factor-alpha (TNF-α), interleukin-1β (IL-1β), IL-6, monocyte chemotactic protein-1 (MCP-1), and IL-1 receptor antagonist (IL-1Ra), released into the supernatant were determined by probing culture supernatants with a multiplex human Luminex panel (Bio-Rad, Hercules, CA), according to the manufacturer’s instructions. Data were analyzed using BioPlex™ 200 (Bio-Rad, Hercules, CA). Results were obtained from 4 to 7 independent experiments (4 to 7 independent donors) performed in duplicate. Statistical analysis was done using GraphPad Prism 4 software (GraphPad). To accommodate donor-to-donor variation, cytokine/chemokine induction values are expressed as a percentage of induction relative to the same donor cells exposed to the same condition without PZA treatment. A *P* value ≤ 0.05 is considered statistically significant.

### Mouse infection, drug treatment and CFU enumeration

Mice were infected with CDC1551 as described previously [22]. In brief, 8–10 week–old, female B6D2F1 mice (Jackson Laboratory, Bar Harbor, ME) were infected with low-dose CDC1551 using an aerosol exposure system (In-Tox Products, Moriarty, NM). The mouse lung bacillary load at three hours post-infection (T = 0) was approximately 2.5 log_10_. Starting on day 14 post-infection, a group of infected animals (n = 2–4 per time point) were treated with PZA (150 mg/kg) 5 days per week by gavage. On day 42 and 63 post-infection, groups of untreated (n = 4–6 per time point) and PZA-treated animals were euthanized by cervical dislocation and organs were harvested for CFU assay and total RNA isolation. Portion of mouse lungs (∼50% total weight) were homogenized in saline, plated on 7H11 Middlebrook agar plates and incubated at 37°C for 4 weeks to enumerate the number of CFU. A group of uninfected mice (n = 4) were included as controls.

### Total RNA isolation

Mouse lung total RNA was isolated from uninfected and CDC1551-infected (untreated or PZA-treated) animals at 42 and 63 days post-infection (n = 2–6 per group) using Trizol reagent as described previously [23]. Briefly, portions of mouse lungs (∼50% of total weight) were homogenized in Trizol reagent (Invitrogen, Carlsbad, CA), extracted with bromo-chloro-propane (BCP) and total RNA was purified using RNeasy mini kit as per the manufacturer’s instructions (Qiagen, Valencia, CA). The purity and integrity of the isolated RNA was assessed by Nanodrop equipment (Nanodrop Instruments, Wilmington, DE).

### Microarray analysis

Lung total RNA from all study mice isolated at 42 and 63 days was used in the microarray experiments as mentioned earlier [22]. RNA samples from each group of mice at 42 and 63 days (n = 2–6 per group) were processed separately for microarray. The comparator groups to identify gene expression changes at each experimental time point are: a) Mtb-infected versus uninfected and b) PZA-treated versus untreated; all mice in the later group were infected by CDC1551. The global gene expression analysis was performed using Genechip Mouse Gene ST 2.0 arrays according to the instructions of the manufacturer (Affymetrix, Santa Clara, CA). Intensity measurement from 35,240 probes was normalized using the Robust Multi-array Average (RMA) method and the filtered expression (CEL) files were analyzed using Partek Genomic Suite software (Partek Inc., St. Louis, MO). We used two-way ANOVA to analyze the background-corrected raw data. Significantly differentially expressed genes (SDEG) were selected based on uncorrected p value of < 0.05. The list of SDEG specific to Mtb infection (relative to uninfected) or PZA treatment (relative to untreated) were further interrogated for networks and pathways using Ingenuity Pathway Analysis software (IPA, Redwood city, CA). The microarray data has been submitted to the GEO database (accession number GSE48027).

### Quantitative real time PCR (qPCR)

The lung total RNA from uninfected, Mtb-infected and PZA-treated or untreated mice at 42 and 63 days post-infection was used in the qPCR experiments as described earlier [24]. For the uninfected (n = 4) and Mtb-infected (n = 4–6) groups, the RNA was pooled before qPCR. For the PZA-treated group, the RNA from individual mice was processed separately. Reverse transcription of total lung RNA (1 µg) to cDNA was performed using AffinityScript cDNA synthesis kit, according to the manufacturer’s protocol (Agilent Technologies, Santa Clara, CA). The cDNA was amplified using Brilliant SYBR Green QPCR Master Mix, according to the manufacturer in a Stratagene Mx3005p instrument (Agilent Technologies, La Jolla, CA). ROX, an inert internal reference dye, was included in all the reactions. The oligonucleotide primers specific to mouse genes of interest were obtained from PrimerBank (www. primerbank.org). The house-keeping, glyceraldehyde-3-phosphate dehydrogenase (*gapdh*) gene was always run in parallel with the target genes and their expression levels were used for normalization. The qPCR data analysis and threshold cycle (Ct) calculations were done using MxPro software (Agilent Technologies, La Jolla, CA) and the fold-change in gene expression was obtained using the formula 2^−ΔΔCt^ (ΔC_t_ is the difference in Ct between the target gene and *GAPDH*; ΔΔCt is the difference between the infected and uninfected or between PZA treated and untreated samples). The qPCR experiment was repeated at least 3 times.

## Results

### Effect of PZA on Cytokine and Chemokine Release from Mtb-infected Human Monocytes

**Figure 1:**
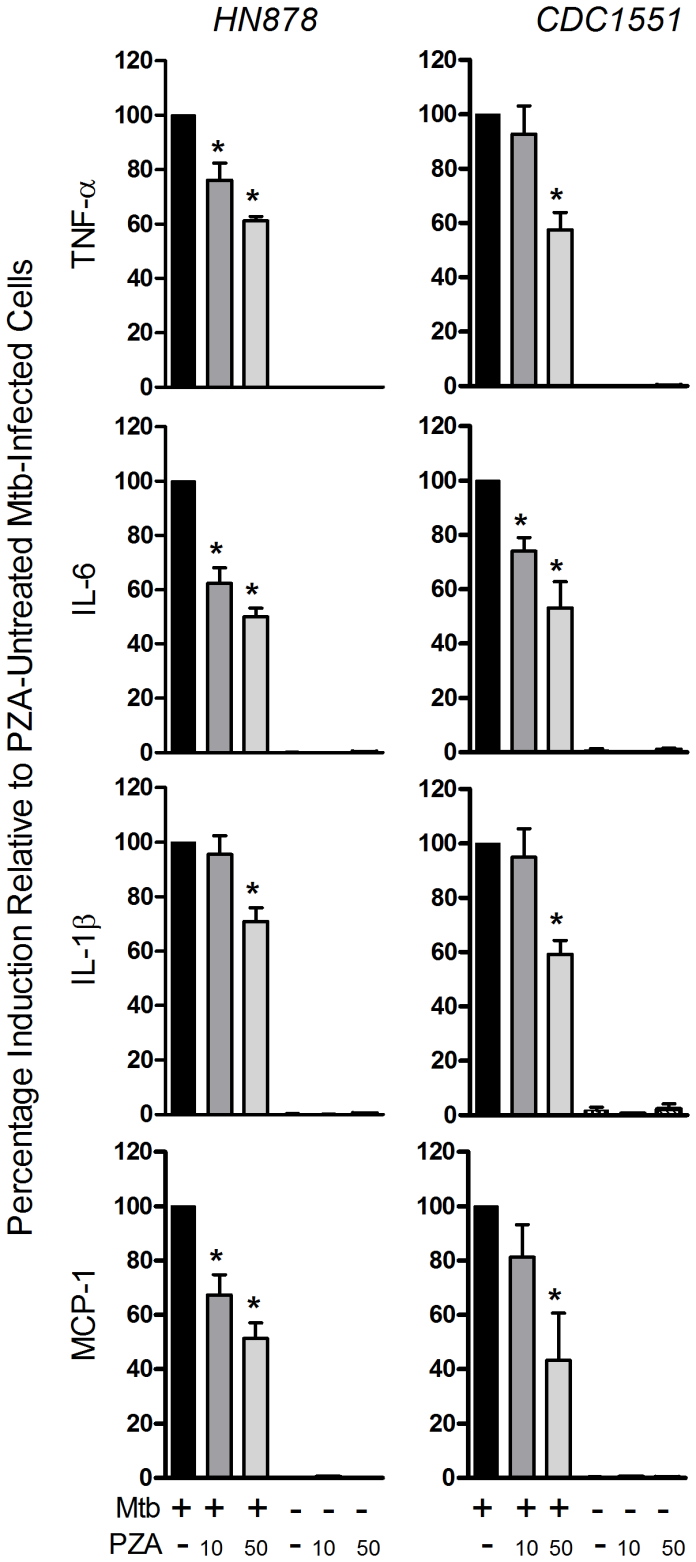
PZA effect on the release of pro-inflammatory cytokines. Human monocytes treated with PZA (10 or 50 µg/ml) and simultaneously infected with Mtb strain CDC1551 or HN878. Pro-inflammatory mediators were measured in culture supernatants at 24 hours post-infection. Data are from seven independent experiments (independent donors; N = 7) performed in duplicate, and presented as percentage induction relative to PZA-untreated Mtb-infected cells ± SD. * statistically significant; *P*≤0.05 compared with PZA-untreated Mtb-infected cells.

In adults the peak serum concentrations associated with the standard dose of PZA treatment have been reported to be in the range of 20 to 50 µg/ml [25]–[28]. However, much higher concentrations (431+/– 220 µg/ml) have been observed in the pulmonary epithelial lining fluid [29]. To determine an appropriate dose of PZA for use in our experiments, we cultured adherent monocytes in vitro in the presence of PZA at concentrations of 10, 20, 100, 200, and 400 µg/ml and determined their viability after 24 hours as measured by trypan blue exclusion. Exposure of human monocytes to concentrations of PZA up to 100 µg/ml resulted in monocyte viability of 97% or better. At higher concentrations, >10% cell mortality was observed. Treatment of monocytes with PZA alone at 10 and 50 µg/ml induced negligible levels (pg/ml) of inflammatory mediators that were comparable to those from cells cultured in media alone. Thus, at these concentrations, PZA alone did not exert significant stimulatory or inhibitory effects on cytokine release by cultured monocytes (Figure 1, Supplementary Figure S1). All subsequent studies were carried out using PZA concentrations of 10 and 50µg/ml. The cytokines measured in the culture supernatants were selected from the panel of cytokines that displayed striking fluctuations in their concentration levels in our previous TB patient study when the anti-mycobacterial treatment shifted from the intensive four-drug phase to the continuation phase [16]. When monocytes were infected with Mtb, followed by immediate treatment with PZA at 10 or 50 µg/ml, dose-dependent reductions in the levels of pro-inflammatory molecules TNF-α, IL-6, IL-1β and MCP-1, relative to infection alone, were observed (Figure 1). While the total amount (pg/ml) of pro-inflammatory mediators released was donor-dependent, the percentage of cytokine produced relative to PZA-untreated Mtb-infected monocytes was consistent among donors and independent of the Mtb strain used. Levels of IL-1Ra were unaffected by PZA treatment and comparable to those induced by Mtb infection alone indicating that the effect of PZA was selective for specific pro-inflammatory cytokines/chemokines (Supplementary Figure S1). Since no difference in CFU was observed between Mtb-infected cells treated with PZA and Mtb-infected cells left untreated the observed reduction in the levels of pro-inflammatory cytokines/chemokines was not attributable to changes in the bacterial load (Table 1). The CFU data were in agreement with previous reports suggesting that PZA does not have antimicrobial activity against Mtb in cultured human and mouse cells [30]–[31].

**Table 1:** Effect of PZA on the growth of CDC1551 and HN878 in human monocytes.

	Colony Forming Units (CFU) *	
Mtb Strain/Condition	*Inoculum*	*Day1*
CDC1551	3.3±1.3	3.0±1.7
CDC1551 + PZA (10 µg/ml)	3.3±1.3	3.0±1.9
CDC1551 + PZA (50 µg/ml)	3.3±1.3	3.0±1.7
HN878	2.3±0.4	3.0±0.3
HN878 + PZA (10 µg/ml)	2.3±0.4	2.9±0.3
HN878 + PZA (50 µg/ml)	2.3±0.4	3.1±0.3

* CFU**×**10^5^ ± SD**.**

### Effect of PZA on Cytokine Release by Human Monocytes Stimulated with Selected TLR Agonists

**Figure 2:**
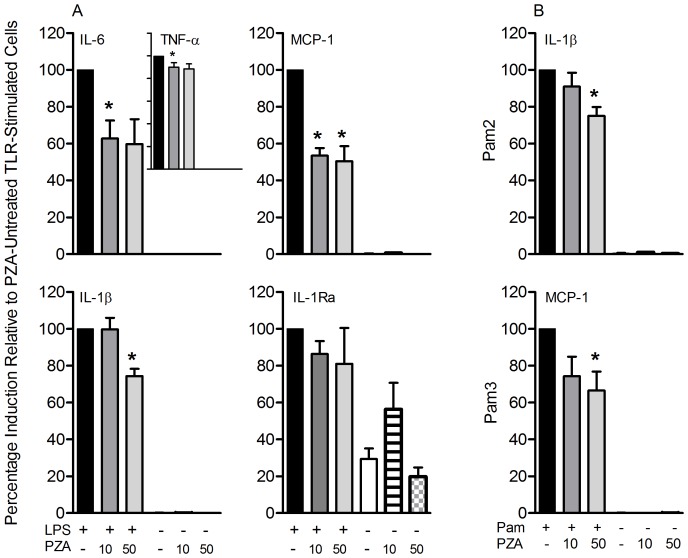
PZA effect on monocytes stimulated with selected TLR agonists . Human monocytes treated with PZA (10 or 50 µg/ml) were simultaneously stimulated with (A) TLR4 (LPS, 100 ng/ml) or (B) TLR2/6 (Pam2, 250 ng/ml) and TLR2/1 (Pam3, 250 ng/ml) agonists. Pro-inflammatory and down-regulatory mediators were measured in the culture supernatants at 24 hours post-stimulation. Data are from 4 – 7 independent experiments (independent donors; N =  4 – 7) performed in duplicate and presented as percentage induction relative to PZA-untreated TLR-stimulated cells ± SD. * statistically significant; *P* ≤ 0.05 compared with the PZA-untreated TLR-stimulated cells.

To confirm that PZA can affect cytokine/chemokine production by monocytes independent of changes in the Mtb bacillary load monocytes were stimulated with TLR agonists and the impact of simultaneous treatment with PZA on the release of specific cytokines/chemokines was evaluated and found to differ among the agonists. When LPS (TLR4 agonist) was used to stimulate monocytes, PZA treatment showed a concentration-dependent inhibitory effect in the release of all four pro-inflammatory mediators tested, and did not alter the release of IL-1Ra, a known IL-1β inhibitor, in Mtb-infected cells (Figure 2A). Our observation that the levels of IL-1Ra were unaffected by PZA treatment suggested that the reduction in the levels of IL-1β was not IL-1Ra-dependent. When PZA-treated cells were simultaneously stimulated with Pam2CSK4 (TLR2/6) or Pam3CSK4 (TLR2/1) agonists and PZA, concentration-dependent inhibition was seen only for IL-1β or MCP-1, respectively (Figure 2B). Thus, the TLR stimulation data confirmed that PZA affected monocyte cytokine/chemokine production independently of Mtb infection or Mtb bacillary load in the monocytes.

### Effect of PZA Treatment on the Lung Transcriptome of Mtb-Infected Mice

**Figure 3:**
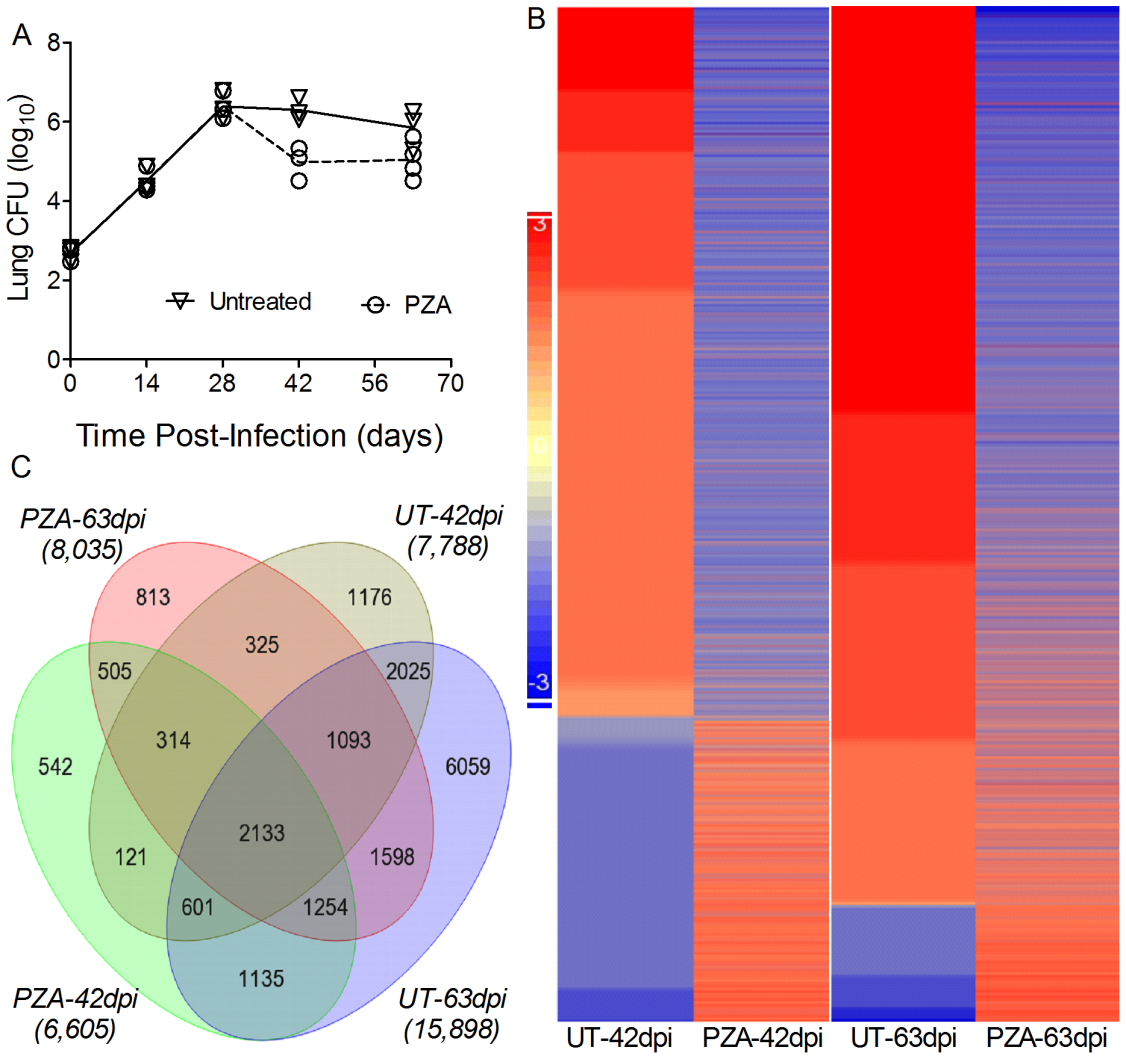
Bacillary load and genome-wide transcriptional analysis of untreated or PZA-treated Mtb-infected mouse lungs. (A). Lung bacillary load in the untreated or PZA-treated Mtb-infected mice (CFU). Statistical analysis between Mtb-infected mice PZA-treated and untreated showed a *P* = 0.0107 at 42 days and *P* = 0.0825 at 63 days post-infection. (B). Intensity map of SDEG in the untreated and PZA-treated Mtb-infected mouse lungs at 42 and 63 days. The scale bar ranges from +3 (up-regulated; red) to -3 (down-regulated; blue). (C). Venn diagram showing the number of SDEG shared between untreated and PZA-treated Mtb-infected mouse lungs at 42 and 63 days. The total number of SDEG in each group is shown in parenthesis.

To extend our in vitro findings, we treated Mtb-infected mice with PZA and analyzed the changes in global gene expression profiles in the lungs at 42 and 63 days post-infection (Figure 3). Both untreated and PZA-treated mice had similar initial numbers of lung bacilli (Figure 3A). PZA treatment was started on day 14 post-infection as previously described [32], [33]. Compared to untreated mice, lung CFU counts in the PZA-treated animals were not significantly different at 28 and 63 days. However, a moderate and transient reduction in CFU was noted in the PZA-treated mice at 42 days (Figure 3A). To determine the changes in host gene expression profile in the PZA-treated mice, we performed microarray analysis using total lung RNA from PZA-treated mice at 42 and 63 days and compared them to the untreated counterpart (Figure 3B and 3C). Relative to the uninfected animals, 7,788 and 15,898 genes were significantly differentially expressed (SDEG; <0.05) in the Mtb-infected mouse lungs at 42 and 63 days, respectively. Of these, about 53% (4,100 genes) and 90% (14,243genes) were up regulated in the Mtb-infected, relative to the uninfected mice at 42 and 63 days, respectively. There were 5,852 SDEG commonly regulated between 42 and 63 days, in the Mtb-infected, relative to uninfected mice. In the PZA-treated animals, expression of 6,605 and 8,035 SDEGs was perturbed at 42 and 63 days, respectively, compared to untreated mice (Figure 3C). Among the SDEG, 3,169 at 42 days and 6,078 at 63 days were commonly regulated between the untreated and PZA-treated mice. Of these SDEG, 67.3% and 65% were down regulated in the lungs of PZA-treated, relative to the untreated, mice at 42 and 63 days, respectively (Figure 3B). There were 4,206 SDEG commonly regulated in the PZA-treated, relative to untreated, mice between 42 and 63 days.

### Confirmation of Microarray Data by qPCR

The qPCR experiments were done using the total RNA isolated from the lungs of uninfected, Mtb-infected and PZA-treated infected mice. We selected 13 mouse genes for qPCR to confirm the direction and level of expression observed in the microarray experiments. The qPCR results for the selected genes were consistent with the direction of expression observed by microarray experiments (Table 2). However, due to the differences in the sensitivity of detection, the absolute expression levels were different between the qPCR and microarray assays. These observations, at the gene expression level, showing that PZA reduced *il1β*, *il6* and *tnfα* support the results seen in vitro in Mtb-infected monocytes at the protein level.

**Table 2:** Expression of selected mouse genes determined by qPCR and microarray.

	42 dpi				63 dpi			
	Untreated		PZA		Untreated		PZA	
Genes	qPCR*	Array	qPCR*	Array	qPCR*	Array	qPCR*	Array
*tnfα*	3.4±1.4	2.5	1.7±1.34	–2	11.8±7.8	11.3	–1.4±0.2	–1.83
*cd14*	47. 2±14.5	1.5	–7.7±1.2	–1.86	33.7±14.8	6.5	–3.2±0.1	–2.1
*cxcl10*	91.9±8.5	25	–18.9±9.0	–2	30.8±14.6	51.7	–5.0±1.2	–2.14
*ifnγ*	137.1±92	2.2	–14.9±4.9	–2.4	7.1±6.4	16.1	–3.5±1.5	–3.4
*cxcl9*	671.5±160.1	32.4	–4.7±0.3	–1.6	474.2±154.1	180.9	–4.5±0.5	–1.81
*il6*	6.6±2.0	1.2	–3.2±0.5	–1.8	4.7±1.2	5.6	–1.3±0.2	–2.1
*il1β*	9.7±3.0	3	–7.8±2.7	–2.6	17.2±5.1	14.84	–10.1±2.1	–3.07
*saa3*	54.8±4.8	15.3	–7.5±0.8	–2.13	20.8±5.1	59.9	–7.6±1.6	–3
*ccl2*	7.3±0.6	2	–3.5 ±0.2	–2.4	16.5±1.5	10.1	–6.9±1.1	–2.3
*ly6A*	3.2±0.4	1.9	–15.0±1.2	nd	1.0±0.3	nd	–5.3±0.3	–1.32
*tlr9*	17.3±4.0	1.6	–1.9±0.2	nd	112.0±38.9	8.86	–10.1±0.1	nd
*gpd2*	1.9±0.4	1.4	nd	–1.2	1.5±0.2	2.89	nd	nd
*fasn*	6.5±0.9	1.5	nd	nd	4.5±1.2	1.9	nd	nd
*mmp2*	3.9±0.5	1.2	1.1±0.3	1.29	2.5±0.3	3.2	nd	–1.7

* qPCR values are average +/– standard error from at least 3 independent experiments; nd-not detected.

### Effect of PZA Treatment on the Expression of Selected Pro-Inflammatory Network Genes in Mtb-Infected Mouse Lungs

We next interrogated the untreated and PZA-treated infected mouse lung microarray data to determine the expression of selected pro-inflammatory network genes regulated by *il1β*, *il6*, *tnfα* and *mcp1* at 42 and 63 days (Table 3). The target genes in these networks are either directly or indirectly regulating and/or regulated by the central regulator genes (i.e., *il1β*, *il6*, *tnfα* and *mcp1*).

**Table 3:** Number of mouse genes in the selected pro-inflammatory networks.

		42 days					63 days					
		*Untreated*		*PZA-treated*			*Untreated*			*PZA-treated*		
Network	Total genes	Up	Down	Up	Down	NS*	Up	Down	NS*	Up	Down	NS*
*IL-1β*	503	381	122	51	282	170	346	31	126	58	274	171
*IL-6*	568	448	120	57	339	172	414	25	129	63	318	187
*TNF-α*	894	663	231	108	465	321	614	56	225	115	457	322
*MCP-1*	158	119	39	17	91	50	104	11	43	17	87	54

* not significant.

As shown in Table 3, higher numbers of genes in the IL-1β, IL-6, TNF-α and MCP-1 networks were significantly up-regulated in the lungs of Mtb-infected, relative to the uninfected mice, at both 42 and 63 days. However, some of the network genes differentially expressed in the untreated mice at 42 days were either not expressed or had insignificant levels of expression at 63 days (Table 3). Importantly, most of the Mtb-infection induced IL-1β, IL-6, TNF-α and MCP-1 network genes were down regulated by PZA-treatment at both 42 and 63 days, while only 8–10% of these pro-inflammatory network genes were up-regulated, in all the four pro-inflammatory networks at both time points. The list of genes in each of the tested pro-inflammatory networks and their level of expression are presented in Supplementary Table S1 and Supplementary Figure S2. Taken together, consistent with our in vitro findings, PZA-treatment significantly dampened the expression of many of the pro-inflammatory network genes in Mtb-infected mouse lungs that are regulated by IL-1β, IL-6, TNF-α and MCP-1.

### Effect of PZA Treatment on the Expression of Lung Inflammatory Response Network Genes in Mtb-Infected Mice

**Figure 4:**
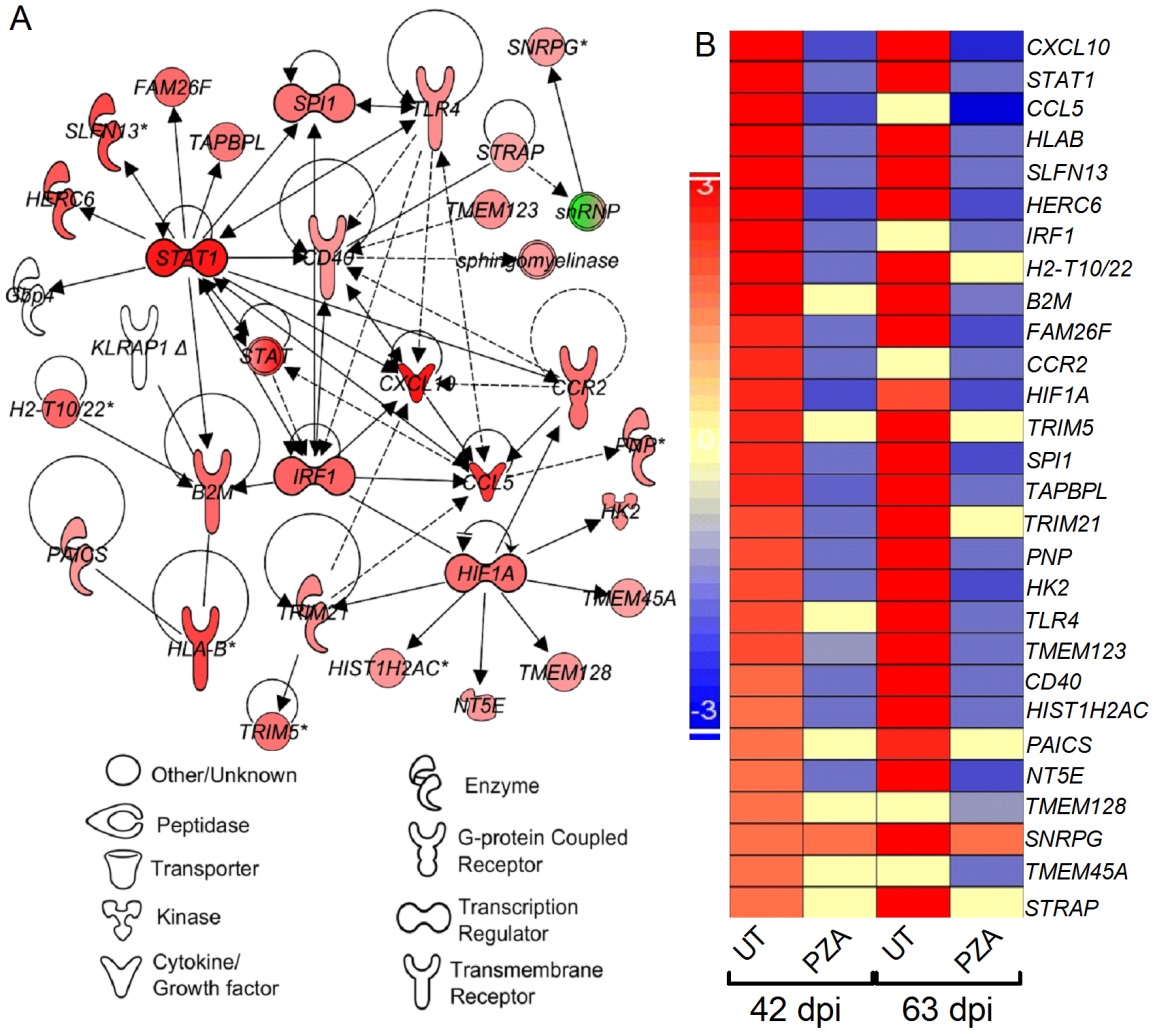
Expression of lung inflammatory response network genes in the untreated or PZA-treated mice. (A). Interaction of genes involved in the host inflammatory response network in the untreated Mtb-infected mouse lungs at 42 days. Red and green symbols in the networks indicate up-, and down-regulated SDEG and the gradation in the color intensity of symbols is proportional to their relative expression levels. (B). Intensity map of 28 SDEG in the untreated and PZA-treated Mtb-infected mouse lungs at 42 and 63 days. The scale bar ranges from +3 (up-regulated; red) to –3 (down-regulated; blue).

Since PZA-treatment of Mtb-infected human primary cells and mouse lungs reduced IL-1β, IL-6, TNF-α and MCP-1 at the protein and gene transcription levels, respectively, we hypothesized that PZA-treatment reduce general lung inflammation in Mtb-infected mice. To address the effect of PZA treatment on the lung inflammation, we analyzed the level and pattern of expression of inflammatory response network genes in mouse lungs at 42 and 63 days and compared to the untreated counterpart (Figure 4). A total of 28 SDEG were involved in the inflammatory response network. These genes encode transcriptional regulators (*stat1*, *irf1*, *spi1* and *hif1a*), cytokines and chemokines (*cxcl10*, *ccl5* and *hk2*), enzymes (*slfn13*, *herc6*, *paics*, *trim21* and *pnp*) and membrane receptors (*tlr4*, *cd40*, *b2m*, *hlab* and *ccr2*). All of these SDEG at 42 days and 23 out of 28 SDEGs at 63 days were up-regulated in the lungs of untreated infected mice (Figure 4B). In contrast, 20 and 21 out of 28 SDEGs in the inflammatory network genes were significantly down-regulated in the PZA-treated, relative to untreated, infected lungs at 42 and 63 days, respectively. The expression levels of *snrpg* was similar between untreated and PZA-treated, while 7 and 5 out of 28 SDEGs were either not expressed or had insignificant levels in the PZA-treated mouse lungs at 42 and 63 days, respectively (Figure 4B).

### Effect of PZA Treatment on the Expression of TLR Signaling Pathway Genes in Mtb-Infected Mouse Lungs

In our in vitro infection studies, TLR-agonists-stimulated monocytes showed differential effects on the production pro-inflammatory cytokines upon PZA treatment. To further understand the effect of PZA treatment on TLR-mediated inflammatory response, we analyzed the canonical TLR signaling pathway in the untreated versus PZA-treated mouse lungs using microarray gene expression (Supplementary Figure S3). Of the 24 SDEG involved in the TLR signaling, expression of 14 and 15 SDEG were up-regulated in untreated, relative to uninfected, mouse lungs at 42 and 63 days, respectively. While 3 SDEG were down-regulated at 42 days, none were down-regulated at 63days in the untreated animals. In addition, expression of 7 and 9 SDEG at 42 and 63 days, respectively, were insignificantly expressed in these animals. Importantly, compared to the untreated animals, PZA-treated mice showed down-regulation of 10 out of 24 SDEG at 42 and 63 days. At these time points, only 2 and 3 SDEG were up-regulated, while 12 and 11 were either not expressed or had insignificant expression levels in the lungs of PZA-treated mice (Supplementary Figure S3B). Taken together, PZA treatment significantly dampened the Mtb infection-induced TLR signaling pathway genes in the mouse lungs compared to the untreated animals.

### PZA-Mediated Anti-Inflammatory Response Involves the PPAR and NF-kB Pathways

**Figure 5:**
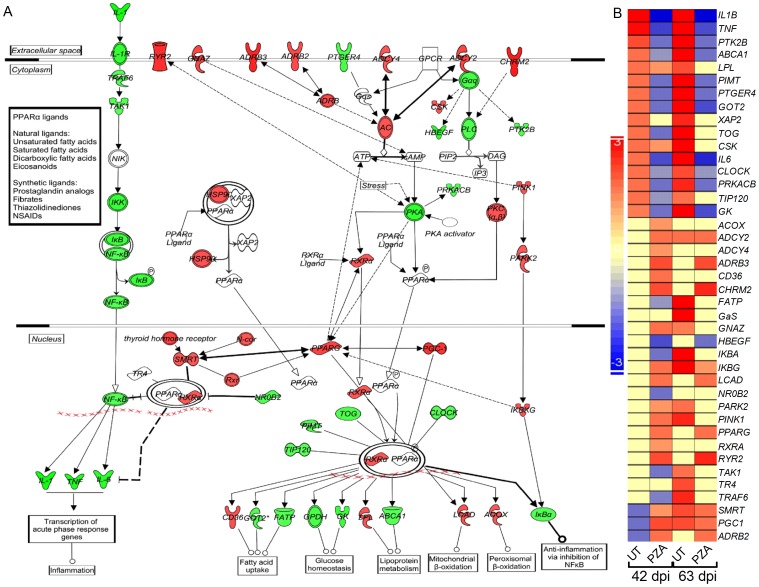
Expression of canonical PPAR and NF-kB pathway genes in the untreated or PZA-treated infected mouse lungs. (A). Canonical PPAR and NF-kB pathway map showing interaction of genes in the untreated Mtb-infected mouse lungs at 42 days. The legends for gene symbols are the same as in Figure 4. Red and green symbols in the networks indicate up-, and down-regulation of SDEG and the gradation in the color intensity of symbols is proportional to their relative expression level. (B). Intensity map of 41 SDEG involved in the PPAR and NF-kB pathways in the untreated and PZA-treated mouse lungs at 42 and 63 days. The scale bar ranges from +3 (up-regulated; red) to –3 (down-regulated; blue).

Since PZA treatment reduced the pro-inflammatory cytokine levels in human monocytes and down-regulated the expression of many genes involved in inflammatory response network and TLR signaling in mouse lungs, we investigated the possible mechanism for the anti-inflammatory activities of PZA. We interrogated the microarray gene expression data from Mtb-infected and PZA treated or untreated mouse lungs at 42 and 63 days to identify the transcriptional regulators (TR) that are significantly differentially expressed in the PZA exposed group. Using the IPA down-stream analysis algorithm, we have identified *pparg* (Peroxisome Proliferator-Activated Receptor, Gamma) as the most significantly differentially expressed, among 36 TR with a regulation z-score value greater than 2. The IPA software uses regulation z-score algorithm to derive functional predictions for the gene expression changes in a dataset. This algorithm is designed in such a way to reduce the chance that random data points will generate significant biological function predictions. We analyzed the expression profile of genes in the PPAR canonical pathway using microarray data from Mtb-infected and PZA treated or untreated mouse lungs at 42 and 63 days post-infection (Figure 5). Of the 41 genes in the PPAR pathway, 16 were up-, and 3 were down-regulated in the Mtb-infected relative to uninfected mouse lungs at 42 days. The number of up-regulated SDEG increased to 29 and none of the 41 SDEGs were down-regulated at 63days. In addition, 22 and 12 genes were either not expressed or had insignificant levels at 42 and 63 days, respectively (Figure 5B). Compared to the untreated infected animal, PZA-treatment up-regulated a larger number of SDEG in the mouse lungs at 42 days (19 versus 16), while equal numbers of SDEG were down-regulated and only 3 were either not expressed or had insignificant levels between these two groups. However, at 63days, 10 SDEG were up-, and 12 were down-regulated, and 19 SDEG were either not expressed or had insignificant levels in the PZA-treated infected mice. In summary, while many of the Mtb-induced genes in the PPAR and in the NF-kB pathways were down-regulated by PZA-treatment, a distinct sub-set of genes (*pparg*, *ryr2*, *adrb3*, *chrm2* and *lcad*) were up-regulated in the treated infected animals at both 42 and 63days (Figure 5B).

## Discussion

In a recent study on the effect of standard DOTS therapy in TB patients we observed an association between the time of withdrawal of PZA and EMB (at the initiation of the continuation phase of TB treatment) and fluctuations in the levels of key cytokines and chemokines measured in the plasma of TB patients [16] (Supplementary Figure S4). We hypothesized that removal of PZA and/or EMB may have contributed to the observed fluctuations. To test our hypothesis, we examined whether PZA, in addition to its well documented anti-mycobacterial properties [34]–[36], also affects the host immune response during treatment of Mtb infection. We observed that PZA treatment of Mtb-infected human monocytes significantly reduces the release of pro-inflammatory cytokines and chemokines, including IL-1β, IL-6, TNF-α and MCP-1. The observed down-regulatory effect exerted by PZA on the release of these pro-inflammatory mediators was independent of bacterial killing, as no changes in the number of CFU were detected between PZA-treated and untreated Mtb-infected monocytes (Table 1). In addition, PZA showed a similar impact on host cytokine production by monocytes stimulated with TLR4 agonist (Figure 2A) in the absence of bacteria. Taken together, our in vitro study indicates that the effect of PZA on monocyte cytokine production is independent of the intracellular bacillary load. In contrast, PZA treatment in mice was associated with about a one log_10_ of Mtb resulting in transient and limited differences in Mtb numbers in the lungs at 42 days (Figure 3A). However, PZA-mediated down-regulation of host gene expression was evident both at 42 days, when there was a significant difference in the CFU between treated and untreated mice and at 63 days, when there was not. PZA-mediated transcriptional down-regulation was observed at both 42 and 63 days post-infection, untreated animals at 63 days post-infection. This suggests that, similar to the in vitro observations, the effect of PZA on the gene expression profile observed in mouse lungs is independent of bacterial killing/load. Consistent with our in vitro observations, PZA treatment down-regulated the expression levels of genes involved in controlling the inflammatory networks, regulated by IL-1β, IL-6, TNF-α and MCP-1.

In addition, the microarray analysis of the mouse lungs revealed that PZA treatment in the context of Mtb infection significantly reduced the levels of expression of genes involved in the regulation of TLR signaling and NF-kB pathways, while up-regulating the levels of expression of genes such as *smrt* (silencing mediator for retinoid or thyroid-hormone receptors; a co-repressor for multiple transcription factor pathways), *adrb2* (adrenoceptor beta 2; a receptor involved in the activation of cyclic adenosine monophosphate; cAMP), adenylate cyclase (*ac*), and *ppar*, all known for their regulatory roles in the inflammatory process (Figure 5). Of particular interest is the up-regulation of *ac*, which encodes Adenylate cyclase responsible for converting adenosine triphosphate (ATP) to cAMP. Increased levels of intracellular cAMP have been reported to down-regulate the production of TNF-α as well as other pro-inflammatory mediators by inhibition of NFkB activation via the cAMP/PKA pathway [37], [38]. We have previously shown that administration of CC-3052, a phosphodiesterase-4 inhibitor (PDE4i), reduces the levels of TNF-α and other inflammatory mediators and improves antibiotic-mediated Mtb clearance in infected mice co-treated with isoniazid [22]. Our present results suggest that similar to our observations with PDE4i, the contribution of PZA to TB therapy may also be derived from its modulation of inflammation via a cAMP-mediated mechanism, in addition to its direct antimicrobial activity [39], [40]. Furthermore, the observed anti-inflammatory activity of PZA may be associated with the previously reported inhibition of pro-inflammatory cytokines by nicotinamide, an analogue of PZA [41], [42] and a potent PDE4i that has been shown to increase the intracellular levels of cAMP [43], [44].

Among the transcriptional regulators affected by PZA treatment of mouse lungs, *PPARG* is the most significant gene that exhibited the highest z-score. PPARs have previously been shown to regulate several genes involved predominantly in lipid metabolism, and more recently their function have been associated with PZA-mediated hepatotoxicity [45], [46]. Three PPAR receptor isotypes have been characterized (PPAR*α, β/δ, γ*) and associated with different disease conditions, including diabetes, cancer and rheumatoid arthritis [47], [48]. PPAR-α and PPAR-γ have been implicated in the regulation of the inflammatory process [49]–[53]. It has been suggested that PPAR-α may dampen inflammation by reducing NF-kB activity [54] and PPAR-γ by inhibiting the NF-kB, STAT-1 and AP-1 pathways [55], [56]. Interestingly, some of the non-steroidal anti-inflammatory drugs used in cancer therapy and in the treatment of rheumatoid arthritis and diabetes have been reported to have PPAR-α or PPAR-*γ* agonist activity [47], [48] (Supplementary Figure S5). Our observations are supported by previous studies on the role of PPAR-γ on Mtb infection in human phagocytes, which showed a link between the PPAR-γ activation and the engagement of TLR and non-TLR pattern recognition receptors in the recognition of virulent Mtb [57], [58]. In addition, the study by Rajaram et al has also shown the involvement of PPAR-γ activation in the down-regulation of the pro-inflammatory mediators [57].

Recognition of Mtb and its antigens by phagocytes via interactions with surface receptors, including the TLRs, leads to the induction of pro-inflammatory mediators such as TNF-α and MCP-1. The fact that PZA modulates the expression of genes in the TLR signaling in Mtb-infected mice and modifies the levels of downstream pro-inflammatory molecules both in vitro and in vivo supports our hypothesis that the drug, by interacting directly with the host immune response, may affect the levels of soluble immune mediators evaluated in the plasma of TB patients. Previous studies have described other host-directed effects of PZA during treatment of microbial infections. Incubation of PZA with *Leishmania major*-infected J774 cells (a murine cell line), and with primary mouse macrophages and bone marrow-derived dendritic cells, induced the up-regulation of activation markers and the release of protective cytokines, suggesting that the drug enhances the host immune response during this parasitic infection [17]. In addition, Kim et al reported that PZA anti-mycobacterial activity was associated with the activation of host cell autophagy, a cell process shown to play a role in regulation of the inflammasome [59]–[61]. Other antimicrobial agents have also been shown to modulate the host immune response independent of their antimicrobial activity [42], [62]–[66]. For example, rifamides have been reported to exert immunosuppressive properties by inhibiting NF-kB activation [67] and RIF has been shown to down-regulate the production of the pro-inflammatory cytokines TNF-α and IL-1β in murine macrophages infected with Mtb and in human monocytes stimulated with LPS or with heat-killed Staphylococci, respectively [63], [68]. In addition, certain quinolones have also been reported to exert anti-inflammatory effects [66]. Moxifloxacin, a powerful second-line anti-Mtb drug, has been shown to inhibit the production of pro-inflammatory cytokines in vitro in human monocytes and in THP1 cells [64], [69]. Moreover, this drug demonstrated a protective anti-inflammatory effect in a murine model of *Candida* pneumonia [70]. Taken together, these observations suggest that the exact nature of the therapeutic regimen used to treat each TB patient may affect the profile of biomarkers of the host immune response detected in the circulation [16]. This could interfere with the utility of host biomarker patterns as a surrogate predictor of response to treatment. Thus, a better characterization and understanding of the interaction of individual antibiotics with the host immune response to Mtb infection will be required to facilitate the identification of biomarkers for clinical use in the management of TB therapy that consistently provide early indications of treatment efficacy or failure.

## Supporting Information

Figure S1 **Effect of PZA on the release of down-regulatory mediator IL-1Ra.** Human monocytes treated with PZA (10 or 50 µg/ml) were simultaneously infected with Mtb strain CDC1551 or HN878. Culture supernatants were analyzed for IL-1Ra levels at 24 hours post-infection. Data are presented as percentage induction relative to PZA-untreated Mtb-infected cells ± SD. * statistically significant; *P* ≤ 0.05 compared with PZA-untreated Mtb-infected cells.
(TIF)

Figure S2 **Intensity map of IL-1β, IL-6, TNF-α and MCP-1 network genes in the untreated or PZA-treated infected mouse lungs.** (A). Level of expression of SDEG regulated by IL-1β. (B). Level of expression of SDEG regulated by IL-6. (C). Level of expression of SDEG regulated by TNF-α. (D). Level of expression of SDEG regulated by MCP-1. The scale bar ranges from +3 (up-regulated; red) to -3 (down-regulated; blue).
(TIF)

Figure S3 **Expression of canonical TLR signaling pathway genes in the untreated or PZA-treated infected mouse lungs.** (A). Canonical TLR signaling pathway map showing interaction of genes in the untreated infected mouse lungs at 42 days. The legends for gene symbols are the same as in Figure 4. Red and green symbols in the networks indicate up-, and down-regulated SDEG and the gradation in the color intensity of symbols is proportional to their relative expression level. (B). Intensity map of 24 SDEG involved in the TLR signaling pathway in the untreated and PZA-treated mouse lungs at 42 and 63 days. The scale bar ranges from +3 (up-regulated; red) to -3 (down-regulated; blue).
(TIF)

Figure S4 **Selected cytokine/chemokine levels in the plasma of HIV infected and uninfected TB patients in response to DOTS treatment.** The horizontal bars denote the phases of antibiotic treatment (dark bar-intensive; light bar-continuation). Note that the evaluation of patient plasma cytokine/chemokine levels continues beyond the completion of DOTS (up to 79 weeks). The dotted vertical line indicates the time (in weeks) of shift in treatment from the intensive to the continuation phase. The values on x- and y- axis are in log_10_ scale.
(TIF)

Figure S5 **Therapeutic drug targets in the canonical PPAR and NF-kB pathway genes.** Canonical PPAR and NF-kB pathway map showing various therapeutic drugs and their target genes in the pathway. The legends for gene symbols are the same as in Figure 4. The expression values shown are from untreated mice lungs at 42 dpi. Red and green symbols in the networks indicate up-, and down-regulation of SDEG and the gradation in the color intensity of symbols is proportional to their relative expression level at 42 dpi.
(TIF)

Table S1 **Expression levels of selected pro-inflammatory network genes** (*in Supplementary Figure S2*) **in the untreated or PZA-treated Mtb-infected mouse lungs**.
(XLS)

## References

[1] WHO (2012) Tuberculosis Fact Sheet. World Health Organization, Geneva, Switzerland

[2] Mitchison D, Davies G (2012) The chemotherapy of tuberculosis: past, present and future. Int J Tuberc Lung Dis 16: 724–732. 22613684 10.5588/ijtld.12.0083 PMC3736084

[3] IUATLD (2000) International Union Against Tuberculosis and Lung Diseases, Paris.

[4] Walzl G, Ronacher K, Djoba Siawaya JF, Dockrell HM (2008) Biomarkers for TB treatment response: challenges and future strategies. J Infect 57: 103–109. 18649943 10.1016/j.jinf.2008.06.007

[5] Holtz TH, Sternberg M, Kammerer S, Laserson KF, Riekstina V, et al (2006) Time to sputum culture conversion in multidrug-resistant tuberculosis: predictors and relationship to treatment outcome. Ann Intern Med 144: 650–659. 16670134 10.7326/0003-4819-144-9-200605020-00008

[6] Wallis RS, Pai M, Menzies D, Doherty TM, Walzl G, et al (2010) Biomarkers and diagnostics for tuberculosis: progress, needs, and translation into practice. Lancet 375: 1920–1937. 20488517 10.1016/S0140-6736(10)60359-5

[7] Benator D, Bhattacharya M, Bozeman L, Burman W, Cantazaro A, et al (2002) Rifapentine and isoniazid once a week versus rifampicin and isoniazid twice a week for treatment of drug-susceptible pulmonary tuberculosis in HIV-negative patients: a randomised clinical trial. Lancet 360: 528–534. 12241657 10.1016/s0140-6736(02)09742-8

[8] Johnson JL, Hadad DJ, Dietze R, Maciel EL, Sewali B, et al (2009) Shortening treatment in adults with noncavitary tuberculosis and 2-month culture conversion. Am J Respir Crit Care Med 180: 558–563. 19542476 10.1164/rccm.200904-0536OC PMC2742745

[9] Sharma SK, Mohan A (2004) Extrapulmonary tuberculosis. Indian J Med Res 120: 316–353. 15520485

[10] De Groote MA, Nahid P, Jarlsberg L, Johnson JL, Weiner M, et al (2013) Elucidating novel serum biomarkers associated with pulmonary tuberculosis treatment. PLoS One 8: e61002. 23637781 10.1371/journal.pone.0061002 PMC3630118

[11] McNerney R, Maeurer M, Abubakar I, Marais B, McHugh TD, et al (2012) Tuberculosis diagnostics and biomarkers: needs, challenges, recent advances, and opportunities. J Infect Dis 205 Suppl 2S147–158. 22496353 10.1093/infdis/jir860

[12] Nahid P, Saukkonen J, Mac Kenzie WR, Johnson JL, Phillips PP, et al (2011) CDC/NIH Workshop. Tuberculosis biomarker and surrogate endpoint research roadmap. Am J Respir Crit Care Med 184: 972–979. 21737585 10.1164/rccm.201105-0827WS PMC3208659

[13] Walzl G, Ronacher K, Hanekom W, Scriba TJ, Zumla A (2011) Immunological biomarkers of tuberculosis. Nat Rev Immunol 11: 343–354. 21475309 10.1038/nri2960

[14] Cliff JM, Lee JS, Constantinou N, Cho JE, Clark TG, et al (2013) Distinct phases of blood gene expression pattern through tuberculosis treatment reflect modulation of the humoral immune response. J Infect Dis 207: 18–29. 22872737 10.1093/infdis/jis499

[15] Djoba Siawaya JF, Beyers N, van Helden P, Walzl G (2009) Differential cytokine secretion and early treatment response in patients with pulmonary tuberculosis. Clin Exp Immunol 156: 69–77. 19196252 10.1111/j.1365-2249.2009.03875.x PMC2673743

[16] Riou C, Perez Peixoto B, Roberts L, Ronacher K, Walzl G, et al (2012) Effect of standard tuberculosis treatment on plasma cytokine levels in patients with active pulmonary tuberculosis. PLoS One 7: e36886. 22606304 10.1371/journal.pone.0036886 PMC3351475

[17] Mendez S, Traslavina R, Hinchman M, Huang L, Green P, et al (2009) The antituberculosis drug pyrazinamide affects the course of cutaneous leishmaniasis in vivo and increases activation of macrophages and dendritic cells. Antimicrob Agents Chemother 53: 5114–5121. 19770283 10.1128/AAC.01146-09 PMC2786368

[18] Sinsimer D, Fallows D, Peixoto B, Krahenbuhl J, Kaplan G, et al (2010) Mycobacterium leprae actively modulates the cytokine response in naive human monocytes. Infect Immun 78: 293–300. 19841079 10.1128/IAI.00816-09 PMC2798203

[19] Manca C, Tsenova L, Barry CE (1999) Mycobacterium tuberculosis CDC1551 induces a more vigorous host response in vivo and in vitro, but is not more virulent than other clinical isolates. J Immunol 162: 6740–6746. 10352293

[20] Manca C, Peixoto B, Malaga W, Guilhot C, Kaplan G (2012) Modulation of the cytokine response in human monocytes by mycobacterium leprae phenolic glycolipid-1. J Interferon Cytokine Res 32: 27–33. 21981546 10.1089/jir.2011.0044 PMC3255513

[21] Sinsimer D, Huet G, Manca C, Tsenova L, Koo MS, et al (2008) The phenolic glycolipid of Mycobacterium tuberculosis differentially modulates the early host cytokine response but does not in itself confer hypervirulence. Infect Immun 76: 3027–3036. 18443098 10.1128/IAI.01663-07 PMC2446685

[22] Koo MS, Manca C, Yang G, O'Brien P, Sung N, et al (2011) Phosphodiesterase 4 inhibition reduces innate immunity and improves isoniazid clearance of Mycobacterium tuberculosis in the lungs of infected mice. PLoS One 6: e17091. 21364878 10.1371/journal.pone.0017091 PMC3045423

[23] Subbian S, Tsenova L, O'Brien P, Yang G, Koo MS, et al (2012) Phosphodiesterase-4 inhibition alters gene expression and improves isoniazid-mediated clearance of Mycobacterium tuberculosis in rabbit lungs. PLoS Pathog 7: e1002262. 10.1371/journal.ppat.1002262 PMC3174258 21949656

[24] Koo MS, Subbian S, Kaplan G (2012) Strain specific transcriptional response in Mycobacterium tuberculosis infected macrophages. Cell Commun Signal 10: 2. 22280836 10.1186/1478-811X-10-2 PMC3317440

[25] Peloquin CA (2002) Therapeutic drug monitoring in the treatment of tuberculosis. Drugs 62: 2169–2183. 12381217 10.2165/00003495-200262150-00001

[26] Heysell SK, Moore JL, Keller SJ, Houpt ER (2010) Therapeutic drug monitoring for slow response to tuberculosis treatment in a state control program, Virginia, USA. Emerg Infect Dis 16: 1546–1553. 20875279 10.3201/eid1610.100374 PMC3294393

[27] Donald PR, Maritz JS, Diacon AH (2012) Pyrazinamide pharmacokinetics and efficacy in adults and children. Tuberculosis (Edinb) 92: 1–8. 21795116 10.1016/j.tube.2011.05.006

[28] Um SW, Lee SW, Kwon SY, Yoon HI, Park KU, et al (2007) Low serum concentrations of anti-tuberculosis drugs and determinants of their serum levels. Int J Tuberc Lung Dis 11: 972–978. 17705974

[29] Conte JE Jr, Golden JA, Duncan S, McKenna E, Zurlinden E (1999) Intrapulmonary concentrations of pyrazinamide. Antimicrob Agents Chemother 43: 1329–1333. 10348747 10.1128/aac.43.6.1329 PMC89273

[30] Heifets L, Higgins M, Simon B (2000) Pyrazinamide is not active against Mycobacterium tuberculosis residing in cultured human monocyte-derived macrophages. Int J Tuberc Lung Dis 4: 491–495. 10864178

[31] Rastogi N, Potar MC, David HL (1988) Pyrazinamide is not effective against intracellularly growing Mycobacterium tuberculosis. Antimicrob Agents Chemother 32: 287. 3129990 10.1128/aac.32.2.287 PMC172157

[32] Ibrahim M, Andries K, Lounis N, Chauffour A, Truffot-Pernot C, et al (2007) Synergistic activity of R207910 combined with pyrazinamide against murine tuberculosis. Antimicrob Agents Chemother 51: 1011–1015. 17178794 10.1128/AAC.00898-06 PMC1803154

[33] Almeida D, Nuermberger E, Tasneen R, Rosenthal I, Tyagi S, et al (2009) Paradoxical effect of isoniazid on the activity of rifampin-pyrazinamide combination in a mouse model of tuberculosis. Antimicrob Agents Chemother 53: 4178–4184. 19620331 10.1128/AAC.00830-09 PMC2764177

[34] McCune RM Jr, McDermott W, Tompsett R (1956) The fate of Mycobacterium tuberculosis in mouse tissues as determined by the microbial enumeration technique. II. The conversion of tuberculous infection to the latent state by the administration of pyrazinamide and a companion drug. J Exp Med 104: 763–802. 13367342 10.1084/jem.104.5.763 PMC2136612

[35] Ahmad Z, Fraig MM, Bisson GP, Nuermberger EL, Grosset JH, et al (2011) Dose-dependent activity of pyrazinamide in animal models of intracellular and extracellular tuberculosis infections. Antimicrob Agents Chemother 55: 1527–1532. 21282447 10.1128/AAC.01524-10 PMC3067197

[36] Zhang Y, Wade MM, Scorpio A, Zhang H, Sun Z (2003) Mode of action of pyrazinamide: disruption of Mycobacterium tuberculosis membrane transport and energetics by pyrazinoic acid. J Antimicrob Chemother 52: 790–795. 14563891 10.1093/jac/dkg446

[37] Zidek Z (1999) Adenosine - cyclic AMP pathways and cytokine expression. Eur Cytokine Netw 10: 319–328. 10477388

[38] Serezani CH, Ballinger MN, Aronoff DM, Peters-Golden M (2008) Cyclic AMP: master regulator of innate immune cell function. Am J Respir Cell Mol Biol 39: 127–132. 18323530 10.1165/rcmb.2008-0091TR PMC2720142

[39] Souness JE, Griffin M, Maslen C, Ebsworth K, Scott LC, et al (1996) Evidence that cyclic AMP phosphodiesterase inhibitors suppress TNF alpha generation from human monocytes by interacting with a 'low-affinity' phosphodiesterase 4 conformer. Br J Pharmacol 118: 649–658. 8762090 10.1111/j.1476-5381.1996.tb15450.x PMC1909726

[40] Souness JE, Aldous D, Sargent C (2000) Immunosuppressive and anti-inflammatory effects of cyclic AMP phosphodiesterase (PDE) type 4 inhibitors. Immunopharmacology 47: 127–162. 10878287 10.1016/s0162-3109(00)00185-5

[41] Zhang Y, Mitchison D (2003) The curious characteristics of pyrazinamide: a review. Int J Tuberc Lung Dis 7: 6–21. 12701830

[42] Ungerstedt JS, Blomback M, Soderstrom T (2003) Nicotinamide is a potent inhibitor of proinflammatory cytokines. Clin Exp Immunol 131: 48–52. 12519385 10.1046/j.1365-2249.2003.02031.x PMC1808598

[43] Campbell PI, Abraham MI, Kempson SA (1989) Increased cAMP in proximal tubules is acute effect of nicotinamide analogues. Am J Physiol 257: F1021–1026. 2557765 10.1152/ajprenal.1989.257.6.F1021

[44] Shimoyama M, Kawai M, Nasu S, Shioji K, Hoshi Y (1975) Inhibition of adenosine 3',5'-monophosphate phosphodiesterase by nicotinamide and its homologues in vitro. Physiol Chem Phys 7: 125–132. 239431

[45] Li AC, Glass CK (2004) PPAR- and LXR-dependent pathways controlling lipid metabolism and the development of atherosclerosis. J Lipid Res 45: 2161–2173. 15489539 10.1194/jlr.R400010-JLR200

[46] Zhang Y, Jiang Z, Su Y, Chen M, Li F, et al.. (2012) Gene expression profiling reveals potential key pathways involved in pyrazinamide-mediated hepatotoxicity in Wistar rats. J Appl Toxicol. 10.1002/jat.2736 22431067

[47] Yamazaki R, Kusunoki N, Matsuzaki T, Hashimoto S, Kawai S (2002) Nonsteroidal anti-inflammatory drugs induce apoptosis in association with activation of peroxisome proliferator-activated receptor gamma in rheumatoid synovial cells. J Pharmacol Exp Ther 302: 18–25. 12065695 10.1124/jpet.302.1.18

[48] Lehmann JM, Lenhard JM, Oliver BB, Ringold GM, Kliewer SA (1997) Peroxisome proliferator-activated receptors alpha and gamma are activated by indomethacin and other non-steroidal anti-inflammatory drugs. J Biol Chem 272: 3406–3410. 9013583 10.1074/jbc.272.6.3406

[49] Delerive P, Fruchart JC, Staels B (2001) Peroxisome proliferator-activated receptors in inflammation control. J Endocrinol 169: 453–459. 11375115 10.1677/joe.0.1690453

[50] Chinetti G, Fruchart JC, Staels B (2000) Peroxisome proliferator-activated receptors (PPARs): nuclear receptors at the crossroads between lipid metabolism and inflammation. Inflamm Res 49: 497–505. 11089900 10.1007/s000110050622

[51] Jiang C, Ting AT, Seed B (1998) PPAR-gamma agonists inhibit production of monocyte inflammatory cytokines. Nature 391: 82–86. 9422509 10.1038/34184

[52] Youssef J, Badr M (2004) Role of Peroxisome Proliferator-Activated Receptors in Inflammation Control. J Biomed Biotechnol 2004: 156–166. 15292582 10.1155/S1110724304308065 PMC551585

[53] Clark RB (2002) The role of PPARs in inflammation and immunity. J Leukoc Biol 71: 388–400. 11867676

[54] Poynter ME, Daynes RA (1998) Peroxisome proliferator-activated receptor alpha activation modulates cellular redox status, represses nuclear factor-kappaB signaling, and reduces inflammatory cytokine production in aging. J Biol Chem 273: 32833–32841. 9830030 10.1074/jbc.273.49.32833

[55] Ricote M, Li AC, Willson TM, Kelly CJ, Glass CK (1998) The peroxisome proliferator-activated receptor-gamma is a negative regulator of macrophage activation. Nature 391: 79–82. 9422508 10.1038/34178

[56] Michalik L, Wahli W (1999) Peroxisome proliferator-activated receptors: three isotypes for a multitude of functions. Curr Opin Biotechnol 10: 564–570. 10600688 10.1016/s0958-1669(99)00030-0

[57] Rajaram MV, Brooks MN, Morris JD, Torrelles JB, Azad AK, et al (2010) Mycobacterium tuberculosis activates human macrophage peroxisome proliferator-activated receptor gamma linking mannose receptor recognition to regulation of immune responses. J Immunol 185: 929–942. 20554962 10.4049/jimmunol.1000866 PMC3014549

[58] Mahajan S, Dkhar HK, Chandra V, Dave S, Nanduri R, et al (2012) Mycobacterium tuberculosis modulates macrophage lipid-sensing nuclear receptors PPARgamma and TR4 for survival. J Immunol 188: 5593–5603. 22544925 10.4049/jimmunol.1103038

[59] Kim JJ, Lee HM, Shin DM, Kim W, Yuk JM, et al (2012) Host cell autophagy activated by antibiotics is required for their effective antimycobacterial drug action. Cell Host Microbe 11: 457–468. 22607799 10.1016/j.chom.2012.03.008

[60] Saitoh T, Fujita N, Jang MH, Uematsu S, Yang BG, et al (2008) Loss of the autophagy protein Atg16L1 enhances endotoxin-induced IL-1beta production. Nature 456: 264–268. 18849965 10.1038/nature07383

[61] Harris J (2011) Autophagy and cytokines. Cytokine 56: 140–144. 21889357 10.1016/j.cyto.2011.08.022

[62] Yuhas Y, Berent E, Ovadiah H, Azoulay I, Ashkenazi S (2006) Rifampin augments cytokine-induced nitric oxide production in human alveolar epithelial cells. Antimicrob Agents Chemother 50: 396–398. 16377722 10.1128/AAC.50.1.396-398.2006 PMC1346828

[63] Ziglam HM, Daniels I, Finch RG (2004) Immunomodulating activity of rifampicin. J Chemother 16: 357–361. 15332711 10.1179/joc.2004.16.4.357

[64] Araujo FG, Slifer TL, Remington JS (2002) Effect of moxifloxacin on secretion of cytokines by human monocytes stimulated with lipopolysaccharide. Clin Microbiol Infect 8: 26–30. 11906497 10.1046/j.1469-0691.2002.00374.x

[65] Melhus A (2001) Effects of amoxicillin on the expression of cytokines during experimental acute otitis media caused by non-typeable Haemophilus influenzae. J Antimicrob Chemother 48: 397–402. 11533005 10.1093/jac/48.3.397

[66] Dalhoff A, Shalit I (2003) Immunomodulatory effects of quinolones. Lancet Infect Dis 3: 359–371. 12781508 10.1016/s1473-3099(03)00658-3

[67] Pahlevan AA, Wright DJ, Bradley L, Smith C, Foxwell BM (2002) Potential of rifamides to inhibit TNF-induced NF-kappaB activation. J Antimicrob Chemother 49: 531–534. 11864954 10.1093/jac/49.3.531

[68] Gil D, Garcia LF, Rojas M (2003) Modulation of macrophage apoptosis by antimycobacterial therapy: physiological role of apoptosis in the control of Mycobacterium tuberculosis. Toxicol Appl Pharmacol 190: 111–119. 12878041 10.1016/s0041-008x(03)00162-5

[69] Weiss T, Shalit I, Blau H, Werber S, Halperin D, et al (2004) Anti-inflammatory effects of moxifloxacin on activated human monocytic cells: inhibition of NF-kappaB and mitogen-activated protein kinase activation and of synthesis of proinflammatory cytokines. Antimicrob Agents Chemother 48: 1974–1982. 15155187 10.1128/AAC.48.6.1974-1982.2004 PMC415605

[70] Shalit I, Horev-Azaria L, Fabian I, Blau H, Kariv N, et al (2002) Immunomodulatory and protective effects of moxifloxacin against Candida albicans-induced bronchopneumonia in mice injected with cyclophosphamide. Antimicrob Agents Chemother 46: 2442–2449. 12121916 10.1128/AAC.46.8.2442-2449.2002 PMC127325

