# Supplementary figures and images for: Host Targeted Activity of Pyrazinamide in Mycobacterium tuberculosis Infection
Source: PLoS One. 2013 Aug 28;8(8):e74082. doi: 10.1371/journal.pone.0074082 (PMC3755974; doi:10.1371/journal.pone.0074082)

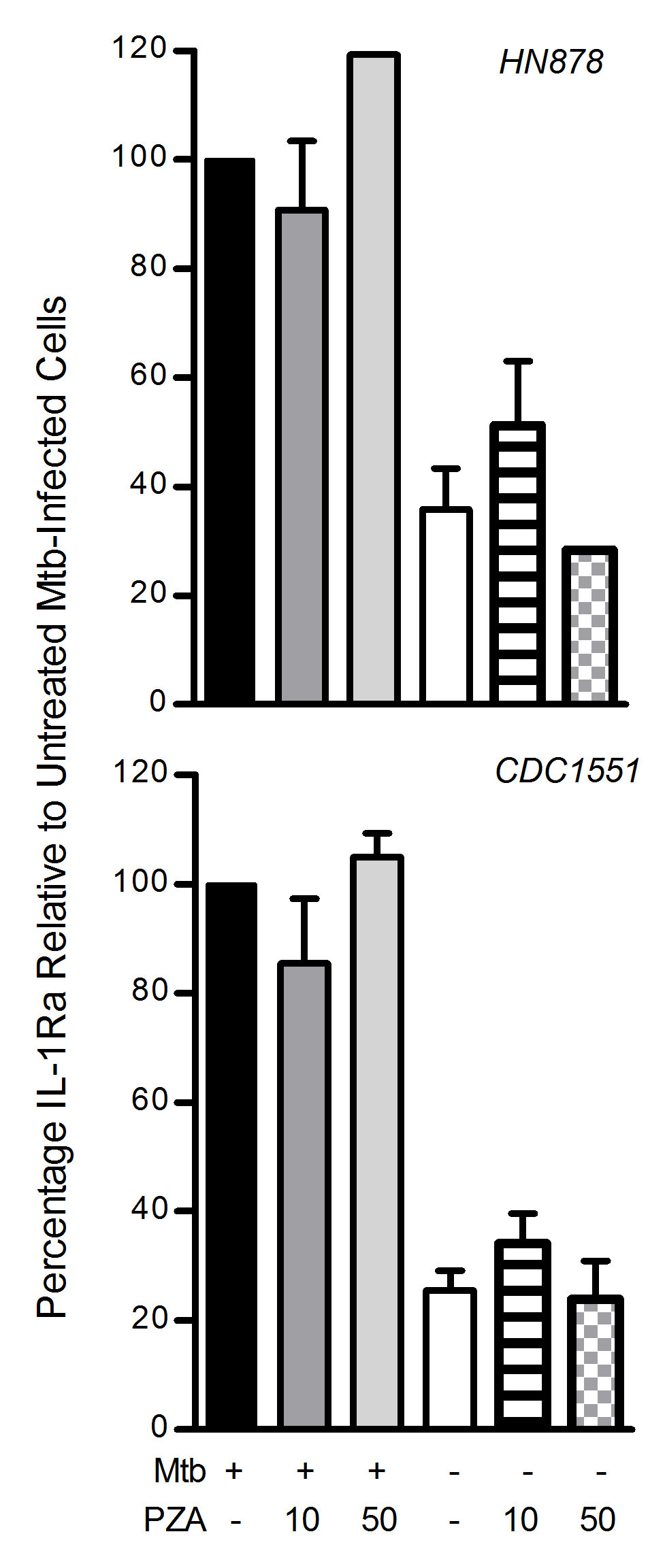

Supplement: Figure S1 — Effect of PZA on the release of down-regulatory mediator IL-1Ra. Human monocytes treated with PZA (10 or 50 µg/ml) were simultaneously infected with Mtb strain CDC1551 or HN878. Culture supernatants were analyzed for IL-1Ra levels at 24 hours post-infection. Data are presented as percentage induction relative to PZA-untreated Mtb-infected cells ± SD. * statistically significant; P ≤ 0.05 compared with PZA-untreated Mtb-infected cells. (TIF) [file pone.0074082.s001.tif]

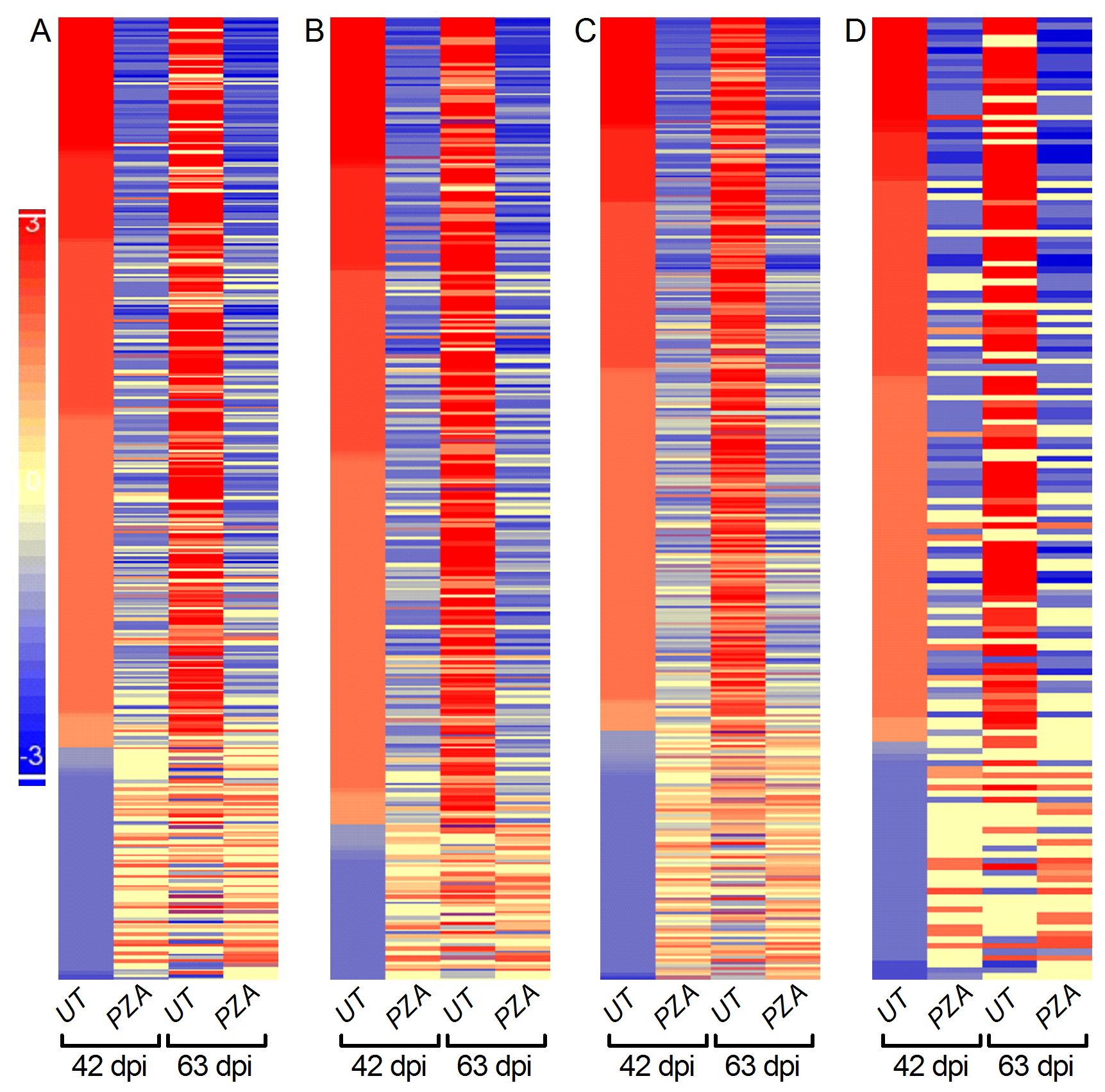

Supplement: Figure S2 — Intensity map of IL-1β, IL-6, TNF-α and MCP-1 network genes in the untreated or PZA-treated infected mouse lungs. (A). Level of expression of SDEG regulated by IL-1β. (B). Level of expression of SDEG regulated by IL-6. (C). Level of expression of SDEG regulated by TNF-α. (D). Level of expression of SDEG regulated by MCP-1. The scale bar ranges from +3 (up-regulated; red) to -3 (down-regulated; blue). (TIF) [file pone.0074082.s002.tif]

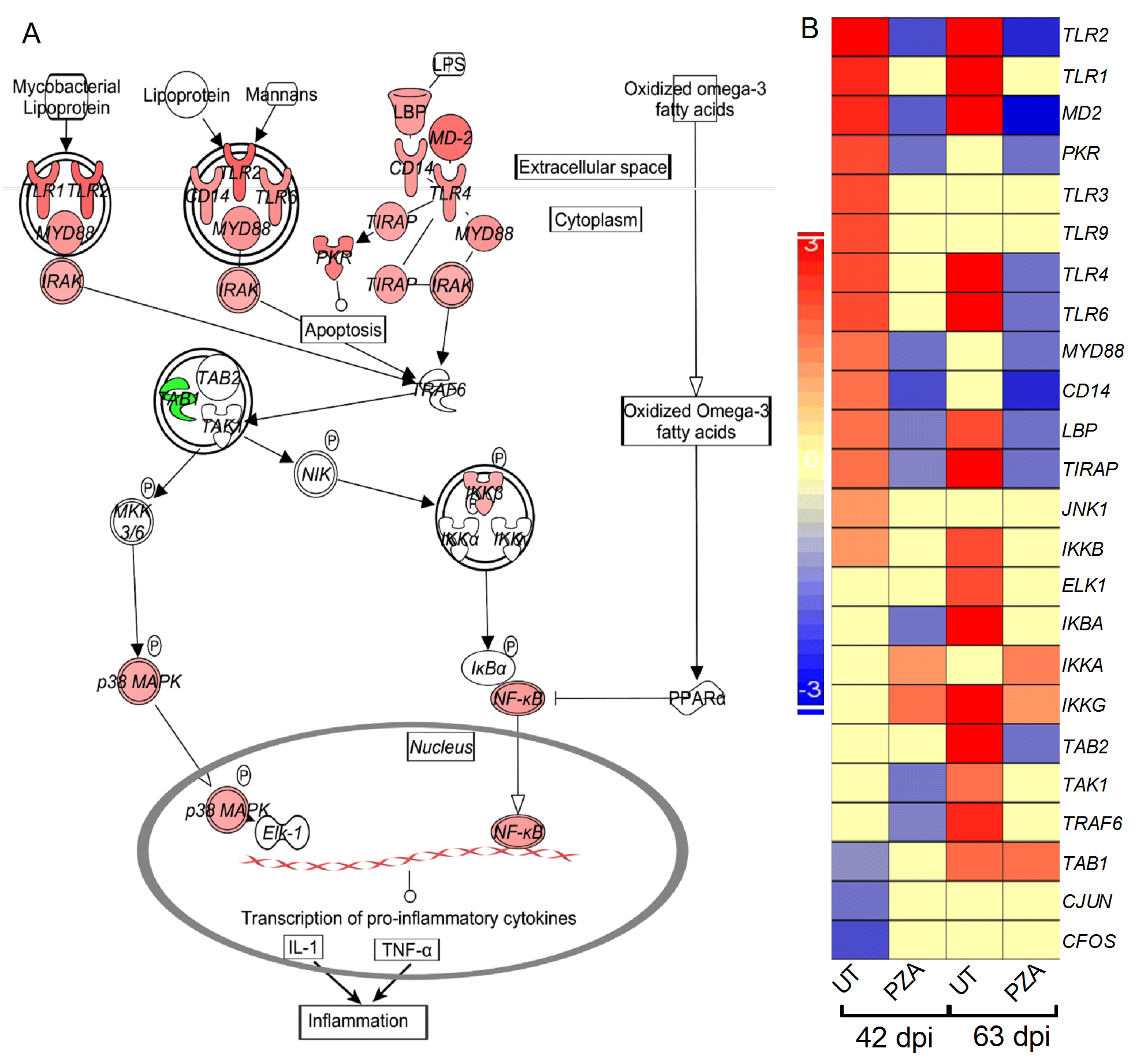

Supplement: Figure S3 — Expression of canonical TLR signaling pathway genes in the untreated or PZA-treated infected mouse lungs. (A). Canonical TLR signaling pathway map showing interaction of genes in the untreated infected mouse lungs at 42 days. The legends for gene symbols are the same as in Figure 4. Red and green symbols in the networks indicate up-, and down-regulated SDEG and the gradation in the color intensity of symbols is proportional to their relative expression level. (B). Intensity map of 24 SDEG involved in the TLR signaling pathway in the untreated and PZA-treated mouse lungs at 42 and 63 days. The scale bar ranges from +3 (up-regulated; red) to -3 (down-regulated; blue). (TIF) [file pone.0074082.s003.tif]

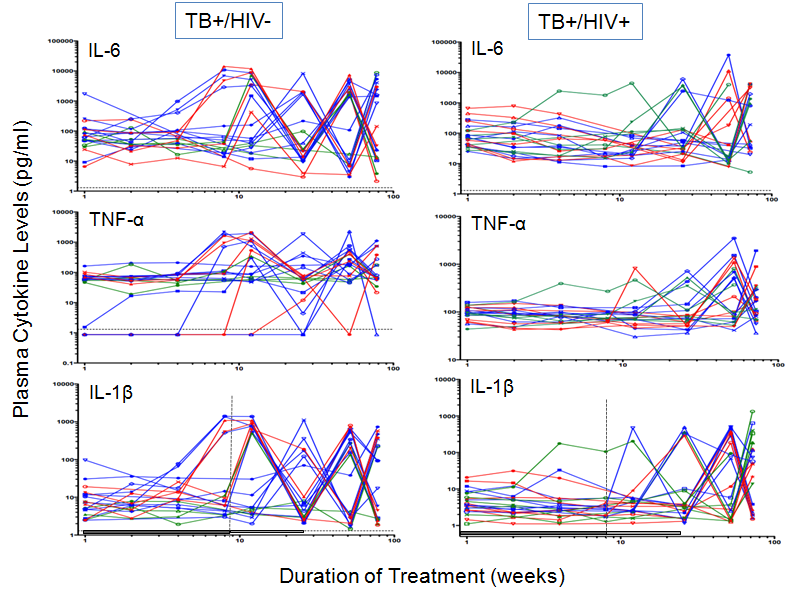

Supplement: Figure S4 — Selected cytokine/chemokine levels in the plasma of HIV infected and uninfected TB patients in response to DOTS treatment. The horizontal bars denote the phases of antibiotic treatment (dark bar-intensive; light bar-continuation). Note that the evaluation of patient plasma cytokine/chemokine levels continues beyond the completion of DOTS (up to 79 weeks). The dotted vertical line indicates the time (in weeks) of shift in treatment from the intensive to the continuation phase. The values on x- and y- axis are in log10 scale. (TIF) [file pone.0074082.s004.tif]

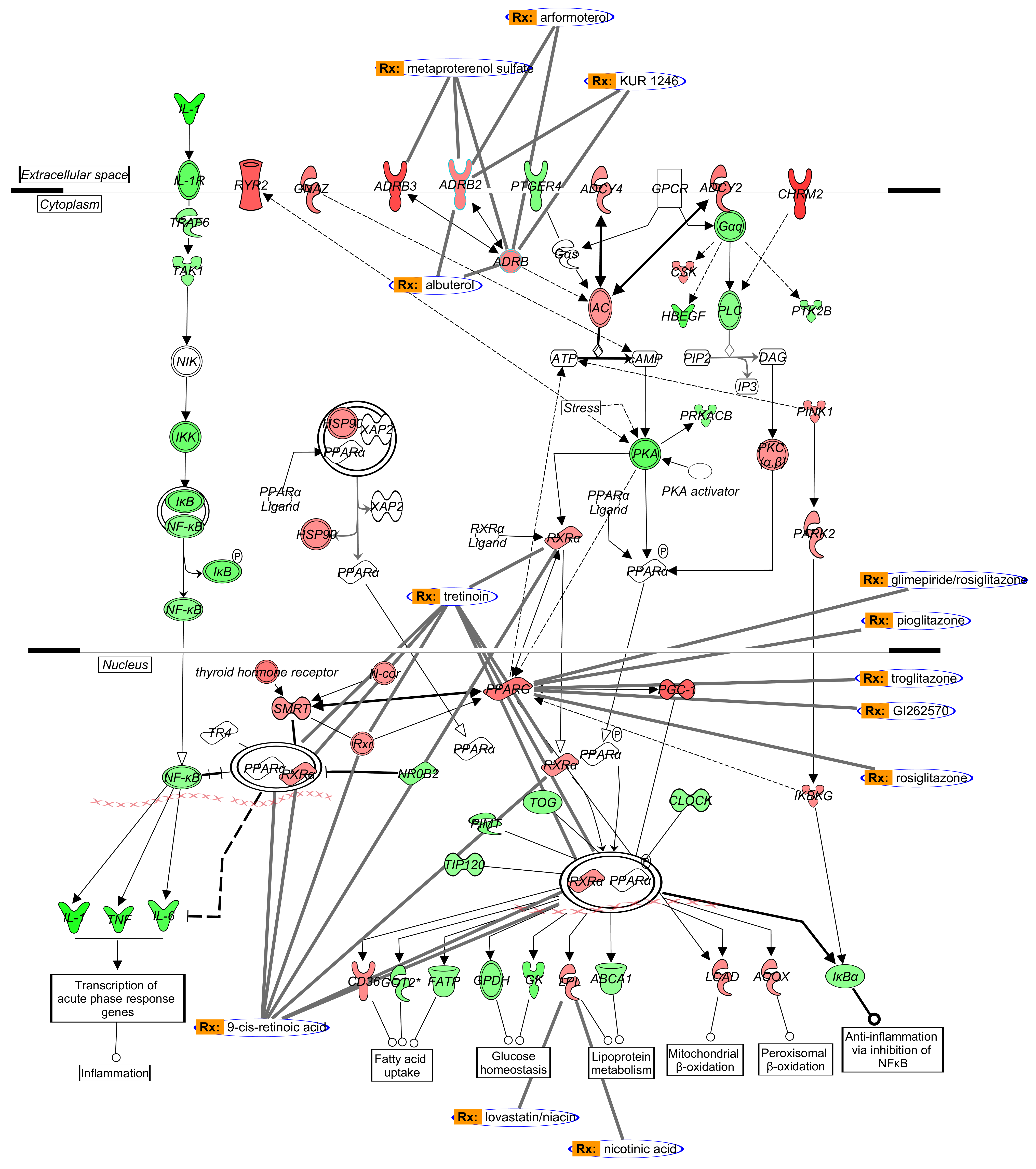

Supplement: Figure S5 — Therapeutic drug targets in the canonical PPAR and NF-kB pathway genes. Canonical PPAR and NF-kB pathway map showing various therapeutic drugs and their target genes in the pathway. The legends for gene symbols are the same as in Figure 4. The expression values shown are from untreated mice lungs at 42 dpi. Red and green symbols in the networks indicate up-, and down-regulation of SDEG and the gradation in the color intensity of symbols is proportional to their relative expression level at 42 dpi. (TIF) [file pone.0074082.s005.tif]
